# High-Voltage Sulfolane Plasticized UV-Curable Gel Polymer Electrolyte

**DOI:** 10.3390/polym11081306

**Published:** 2019-08-04

**Authors:** Shiqi Wang, Chun Wei, Wenwen Ding, Linmin Zou, Yongyang Gong, Yuanli Liu, Limin Zang, Xu Xu

**Affiliations:** 1 College of Materials Science and Engineering, Guilin University of Technology, Guilin 541004, China; 2 Key Laboratory of New Processing Technology for Nonferrous Metals and Materials, Ministry of Education, Guilin 541004, China

**Keywords:** lithium battery, sulfolane, gel polymer electrolyte, high-voltage electrolyte, UV curable

## Abstract

A high-voltage electrolyte can match high-voltage positive electrode material to fully exert its capacity. In this research, a sulfolane plasticized polymer electrolyte was prepared by in situ photocuring. First, the effect of the sulfolane content on the ionic conductivity of the gel polymer electrolyte was investigated. Results showed that the ionic conductivity variation trend was in good agreement with the exponential function model for curve fitting. Second, the activation energy was calculated from the results of the variable temperature conductivity tests. The activation energy was inversely proportional to the sulfolane content. For the sulfolane content of 80 wt. % in gel polymer electrolyte (GPE)-80 (19.5 kJ/mol), the activation energy was close to conventional liquid electrolyte (9.5 kJ/mol), and the conductivity and electrochemical window were 0.64 mS/cm and 5.86 V, respectively. The battery cycle performance test showed that the initial specific discharge capacities of GPE-80 and liquid electrolyte were 176.8 and 148.3 mAh/g, respectively. After 80 cycles, the discharge capacities of GPE-80 and liquid electrolyte were 115.8 and 41.1 mAh/g, and the capacity retention rates were 65.5% and 27.7%, respectively; indicating that GPE-80 has a better specific discharge capacity and cycling performance than the liquid electrolyte. SEM images indicated that GPE-80 can suppress the growth of lithium dendrites. The EDS test showed that GPE-80 can inhibit the dissolution of metal ions in the cathode material.

## 1. Introduction

The lithium-ion batteries are being widely used, because of the growing requirement for mobile phones, electric vehicles and drones [1,2,3]. However, the traditional lithium-ion battery has a low capacity and cannot meet long-life application demands. Therefore, there is an urgent need to develop a battery with a higher specific capacity [4]. Generally, to greatly increase the specific capacity of a battery, it is necessary to develop positive and negative materials. For example, synthetic, higher capacity positive and negative materials increase the use voltage of the positive electrode material, and the use of lithium metal or silicon negative electrodes.

The NCM (Ni-rich transition metal oxide) cathode material has a high specific capacity (>200 mAh/g) and is typically used at a voltage of 4.3 V [5,6]. Further increasing the use voltage of this positive electrode material can increase its energy density and power density. However, a conventional liquid electrolyte has a low electrochemical window, usually less than 4.5 V [7]. Therefore, it is necessary to develop a higher voltage electrolyte to match the cathode material.

Conventional liquid electrolytes have high conductivity and low cost, but their low boiling point, volatility, flammability and explosivity are fatal defects [8,9]. Therefore, it is desirable to develop a high boiling point, highly stability electrolyte material to replace them. Solid and gel electrolytes have become the main research directions [10,11]. However, the presence of a solid electrolyte with low conductivity makes it difficult for operations at room temperature, and the electrolyte and the active material have poor interfacial contact [11]. A gel polymer electrolyte (GPE) has a conductivity comparable to that of a liquid electrolyte and has safety features similar to those of a solid electrolyte, making gel electrolytes the preferred choice for current battery manufacturers [12]. 

Researchers have conducted research on high-voltage gel electrolytes [9,13]. Succinonitrile has a high oxidation potential and has been extensively studied [14,15,16]. Lin et al. studied a succinonitrile plasticized PEGDA (Poly(ethylene glycol) diacrylate) photopolymer gel electrolyte, which is a gel electrolyte with a modulus of 0.43 GPa and an ionic conductivity of 0.6 mS/cm (30 °C) after in situ photocuring [17]. Lv et al. prepared succinonitrile plasticized polyurethane acrylate (PUA) with a conductivity of 1.63 × 10^−3^ S/cm and an oxidation potential reaching 5.3 V using a photopolymer gel electrolyte [18]. Li et al researched a dual-salt LiTFSI/LiBOB polymer electrolyte plasticized by glutaronitrile [19]. The ionic conductivity of the polymer electrolyte reached over 1.0 mS/cm (30 °C), and the oxidation potential was above 5 V.

Sulfolane is a well-known high-voltage electrolyte additive with a high dielectric constant (43.4) and high oxidation potential (>6.35 V) [20]. Cui et al. researched and used this electrolyte with a high-voltage cathode material LiNi_0.5_Mn_1.5_O_4_ (4.7 V) and achieved excellent cycling performance [20]. The oxidation potential of the sulfolane-based electrolyte was above 5.5 V. Li et al. prepared a LiBOB/sulfolane/diethyl carbonate electrolyte that showed an excellent oxidation potential (>5.3 V) and high conductivity. The above studies show that sulfolane is a good high-voltage electrolyte material [21].

However, to the best of our knowledge, the use of sulfolane for in situ curing of plasticized polymer electrolytes has not been reported. Additionally, the conductivity and electrochemical window of sulfolane on polymers requires further research.

## 2. Materials and Methods

### 2.1. Materials

PEGDA600 (*M*_w_ = 600), LiDFOB (lithium difluoro (oxalate) borate), Sulfolane, and NMP (1-methyl-2-pyrrolidone) were analytically pure and purchased from Shanghai Aladdin Biochemical Technology Co., Ltd. (Shanghai, China). Irgacure 819; NCM811 (Li[Ni_0.8_Co_0.1_Mn_0.1_]O_2_ cathode material), carbon black and PVDF were purchased from Xinghua Bennot Battery Material Co., Ltd. (Jiangsu, China). A 100 µm thick FEP (fluorinated ethylene propylene) film was purchased from Suzhou Zeyou Fluorine Plastic Material Technology Co., Ltd. (Suzhou, China). A UV lamp (365 nm LED, 30 W) was purchased from Zhongshan Yanxizhao Lighting Electric Appliance Factory (Zhongshan, China).

### 2.2. Methods

#### Preparation of GPE

In a typical fabrication process for the preparation of an GPE film, PEGDA600, LiDFOB, sulfolane, and Irgacure 819 were mixed, added to a brown bottle, and stirred for 6 h to dissolve. A 100 μm transparent FEP film was placed on a flat glass plate, and a PET film with a thickness of 90 μm and a hole diameter of 18 mm was placed on top. Then, 2 drops of an electrolytic solution were dropped into the hole, covered with a 100 μm FEP film, and pressed with a flat glass plate to extrude excess electrolyte. Curing was then carried out by irradiating the sample with a 365 nm UV lamp for 10 min. The GPE film was obtained by peeling off the film from the mold.

The sulfolane content was controlled to be 0, 20, 40, 60, and 80 wt. %, and the concentration of LiDFOB was fixed at 10.7 wt. % (1.02 mol/L). Different GPEs were obtained, which were named GPE-0, GPE-20, GPE-40, GPE-60, and GPE-80, respectively.

NCM811 and carbon black were mixed, added to the NMP-dissolved in PVDF, to form a solution with a solid content of 45 wt. %, and stirred for 5 h to achieve uniform mixture (NCM811:C65:PVDF = 95:4:1). Then, the samples were coated on aluminum foil and dried in a 80 °C vacuum oven for 24 h to obtain a positive electrode sheet. 

**Figure 1:**
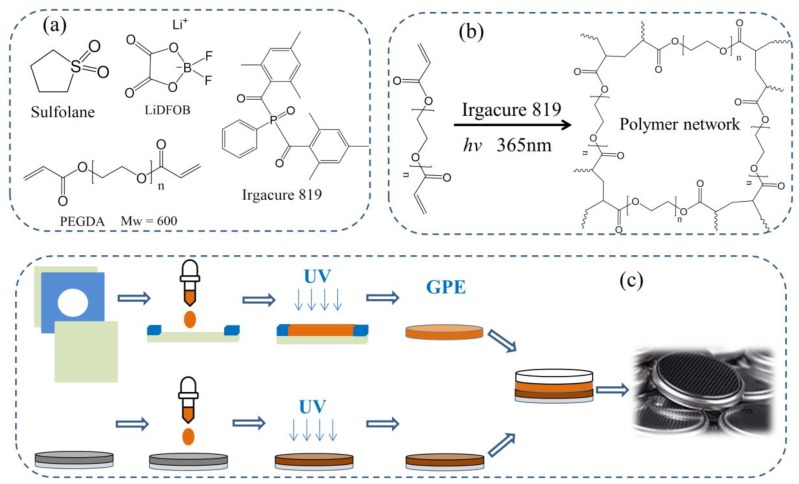
(**a**) Chemical structure of major chemicals; (**b**) Reaction mechanism and possible gel structure of the gel polymer electrolyte (GPE); (**c**) Flow chart of preparation of UV curing a GPE battery.

The electrolyte was dropped onto a 16 mm diameter NCM811 electrode sheet, covered with a GPE film with a diameter of 18 mm, and allowed to sit in the dark for 10 min. Then, the samples were subjected to UV light and cured for 10 min. A 16 mm lithium piece was placed and packaged in the battery case of a CR2025. The battery preparation process is shown in Figure 1.

A 1.1 mol/L nonaqueous solution of LiPF6 (EC:DMC:FEC = 47.5:47.5:5) electrolyte was used for comparison as the liquid electrolyte. 

### 2.3. Characterizations

Fourier transform infrared spectroscopy (FTIR): The powder sample was ground with KBr and then pressed into a thin wafer for testing. Liquid samples were dropped onto a transparent KBr sheet for testing. Test was carried out on a NICOLETNEXUS470-type spectrometer (Perkin-Elmer Company, Waltham, MA, USA). The wavelengths were in the range of 4000–400 cm^−1^, and the resolution was 4 cm^−1^.

Field emission scanning electron microscopy (SEM) images: The sample was placed directly on the S-4800-type instrument (HITACHI Company, Tokyo, Japan) for testing (Acceleration voltage 5.0 kV). In the glove box, the sample was cut and adhered on the copper column with conductive adhesive. Then, it was sealed with a PE bag and quickly transferred to a test chamber of the SEM for testing. In order to prevent the oxidation of the lithium sheet happen.

Energy dispersive spectroscopy (EDS): The test was performed using an X-max type EDS instrument (HORIBA Company, Kyoto, Japan) mounted on an S-4800 SEM. Preparation method of samples were consistent with the SEM test.

Typically, electrochemical characterization was conducted with a CHI660E type electrochemical workstation (CH Instruments, Shanghai, China) at 30 °C.

Ionic conductivity: Electrochemical impedance spectroscopy (EIS) measurements were used to calculate the conductivity of GPEs. The frequency was set to 1 Hz–100 kHz with an amplitude of 5 mV. The sample was prepared as a stainless steel/GPE/stainless steel sandwich configuration for testing. The ionic conductivity (σ) was be calculated by the Equation (1):

(1) $$\sigma =\frac{d}{R_{b}\times S}$$

where *d* and *S* are the thickness and the effective area of the GPE, respectively, and *R_b_* is the bulk impedance of the electrolyte film [19]. Fitting the impedance spectroscopy obtains the intersection of the straight line and the *Z’* axis, which is the value of *R_b_*. The impedance spectrum test data can be found in the supporting information (in Figure S1, Supplementary Materials). The ionic conductivity at different temperatures was tested by change the temperature from 30 to 90 °C; the sample was kept in the incubator for at least 30 min before testing, and results are shown in Figure S2 (Supplementary Materials).

Electrochemical window test: Measurements were carried out using the SS|GPE|Li type cell configuration at 30 °C. The potential range was from −0.5 to 8.0 V with a sweeping rate of 2 mV/s. Cycle performance of battery: The charge/discharge cycle stability test was performed using a BST-4800 type eight-channel battery analyzer (5 V, 50 mA; NEWARE, Shenzhen, China) with a potential window of 2.75–4.3 V (versus Li/Li^+^). The long cycle current density was set to 0.1 C (with respect to the capacity of NCM811 of 180 mAh/g). The rate performance test was carried out through different charge/discharge current densities, and the maximum specific capacity given by the manufacturer is 180 mAh/g. 

## 3. Results and Discussion

### 3.1. Detecting of UV-Cured GPE

**Figure 2:**
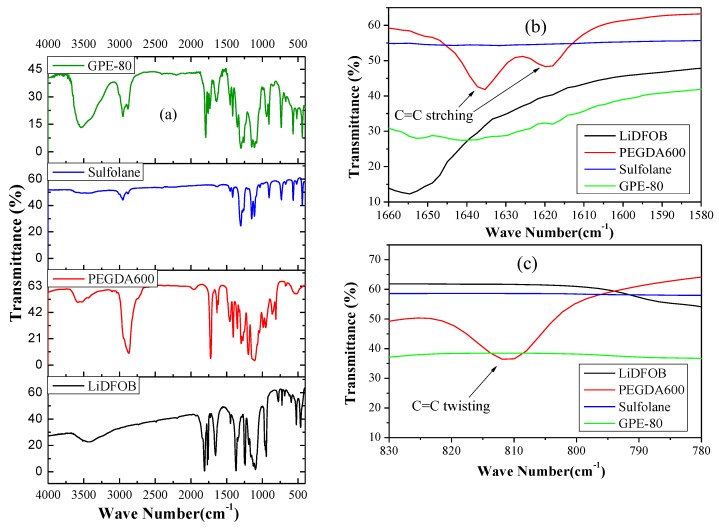
(**a**) FTIR results of raw materials and GPE-80, (**b**) Enlargement of 1660—1580 cm^−1^. (**c**) Amplification of 830—780 cm^−1^.

In order to detect the successful photopolymerization from the chemical structure, we performed an FTIR test, and the results are shown in Figure 2. As shown in Figure 2a, the absorption peak at 1732 cm^−1^ is the C=O stretching absorption of the acetate group of PEGDA [22,23]. The absorption peaks at 1634 and 810 cm^−1^ are vibration and the twisting vibration absorptions of the C=C stretching of the acrylate groups, respectively [23,24]. Comparison of Figure 2b,c shows that the characteristic peak of the C=C double bond disappears after photopolymerization. This indicates successful and complete photopolymerization.

### 3.2. Effect of Sulfolane Content on GPE

**Figure 3:**
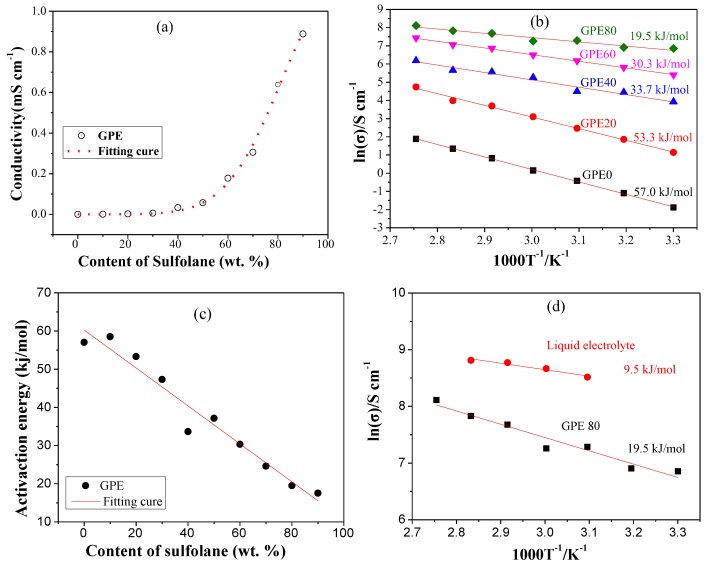
(**a**) The ionic conductivity of GPE changes with the content of sulfolane at 30 °C; (**b**) the relation between Log(σ) and 1/T of GPE; (**c**) change of activation energy with sulfolane content; and (**d**) the relation between Log(σ) and 1/T of liquid electrolyte.

In order to research the effect of sulfolane content on the ionic conductivity, we fixed the lithium salt content at 10.7 wt. % (1.02 mol/L). GPEs containing 0 to 90 wt. % of sulfolane were prepared. Their conductivity was tested at 30 °C (in Figure S1, Supplementary Materials). The scatter plot of the content of sulfolane and the ionic conductivity of the corresponding GPEs were obtained, and are shown in Figure 3a. Through the curve fitting, we found that the variation trend was in good agreement with the exponential function model, and the fitting result is shown in Figure 3a. The R^2^ of the fitting result reached 0.9959, indicating that it has a good correlation. The change trend is represented by the function in Equation (2).

(2) $$\sigma =\exp\left(-10.989+21.48\;c-10.44\;c^{2}\right)$$

where σ is the ionic conductivity of the GPE at 30 °C, in mS/cm. ***c*** is the content of sulfolane, in wt. %. Clearly, the ionic conductivity of GPE increases faster when the sulfolane content is higher than 50%. The results show that when the content of sulfolane is 70, 80, and 90 wt. %, the ionic conductivity of the gel electrolyte reaches 0.31, 0.64, and 0.89 mS/cm, respectively. However, when the sulfolane content is 90 wt. %, the strength of the gel electrolyte is too low to be handled. Therefore, we chose GPE-80, with a sulfolane content of 80 wt. % for additional studies.

The activation energy can reflect the migration difficulty of lithium ion [25]. Generally, σ of the GPE is a Arrhenius-type relationship with the temperature [19,24,26]. The relationship between Log (σ) and 1/T is in accordance with the function in Equation (3) [13].

(3) $$\sigma =\sigma_{0}\exp\left(-\frac{E_{a}}{kT}\right)$$

where *k* is the Boltzmann constant, equal to 1.3806505(24) × 10^−23^ (J/K). T is the temperature of the sample in K. σ is the ionic conductivity of the GPE. *E_a_* is the activation energy; it can be computed from the slope of log(σ) = f(1/T) curve obtained by linear fitting [13]. 

Ionic conductivity of GPEs under variable temperature were tested and results are shown in Figure S2 (Supplementary Materials). The fitting and calculation results are shown in Figure 3b. The results show that the activation energies of GPE-0, GPE-20, GPE-40, GPE-60, and GPE-80 are 57.0, 53.3, 33.7, 30.3 and 19.5 kJ/mol, respectively. Obviously, the activation energy of GPE decreases with the increase of sulfolane. In order to further find the relationship between activation energy and the content of sulfolane, we plotted it as a scatter plot as shown in Figure 3c. Through linear fitting, it can be concluded that the activation energy is inversely proportional to the content of sulfolane. The lower activation energy indicates that the migration of Li^+^ becomes easier. This is consistent with the change in ionic conductivity of GPE. 

The activation energy of the liquid electrolyte and the comparison with CPE-80 are shown in Figure 3d. Results show that the activation energy of GPE-80 (19.5 kJ/mol) is closer to that of conventional liquid electrolyte (9.5 kJ/mol).

**Figure 4:**
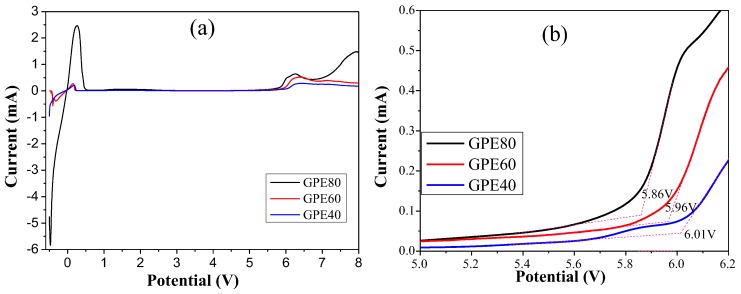
(**a**) Electrochemical window test results of GPE; (**b**) Enlargement of (**a**) at 5.0–6.2 V.

The electrochemical window test results of different GPEs are shown in Figure 4. The results show that the oxidation potentials of GPE-40, GPE-60, and GPE-80 are 6.01, 5.96, and 5.86 V, respectively. The electrochemical window decreases slightly with increasing sulfolane content. However, they all have high electrochemical stability potential and can be used on high-voltage cathode materials.

**Figure 5:**
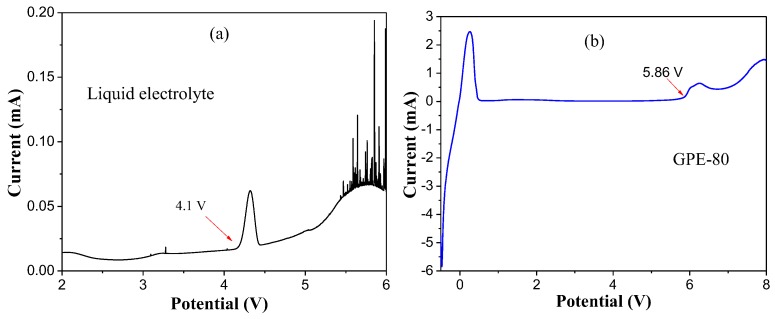
Electrochemical window test results of liquid electrolyte (**a**) and GPE-80 (**b**).

Figure 5a,b shows the electrochemical window test results of liquid electrolyte and GPE-80. The results show that the oxidation potentials of liquid electrolyte and GPE-80 are 4.1 and 5.8 V, respectively. Obviously, the oxidation potential of the conventional GPE-80 is much larger than that of liquid electrolyte. Such a high oxidation potential allows it to match most existing high voltage cathode materials.

### 3.3. Lithium Metal Battery Performance

**Figure 6:**
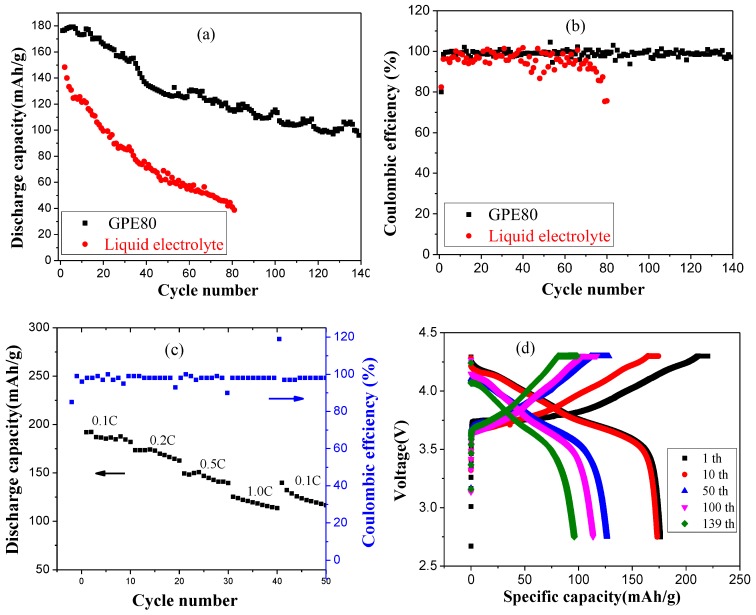
Characterization of lithium battery performance of GPE-80 and liquid electrolyte: (**a**) Cycle performance; (**b**) coulombic efficiency; (**c**) rate performance; and (**d**) charge and discharge profiles of GPE-80 at different cycles.

Taking NCM811 as the cathode material, the battery cycling performances of GPE-80 and liquid electrolyte were compared, and the results are shown in Figure 6. The changes of the specific discharge capacity of the battery under different charge and discharge cycles are shown in Figure 6a. The results show that the initial specific discharge capacities of GPE-80 and liquid electrolyte are 176.8 and 148.3 mAh/g, respectively. After 80 cycles, the discharge capacities of GPE-80 and liquid electrolyte were 115.8 and 41.1 mAh/g, and the capacity retention rates were 65.5% and 27.7%, respectively. Obviously, GPE-80 has better specific discharge capacity and cycle performance than liquid electrolyte. After 140 cycles, the specific discharge capacity of GPE-80 maintains at 96.1 mAh/g, and the capacity retention rate reached 54.3%. 

Figure 6b shows the coulombic efficiency test results of GPE-80 and liquid electrolyte. The initial coulombic efficiency of GPE-80 and the liquid electrolyte are 80.1% and 82.4%, respectively. However, it can be seen from the results that the change in the coulombic efficiency of the liquid electrolyte is larger than that of GPE-80 during cycling. Through 80 cycles, the coulombic efficiency of the liquid electrolyte decreased to 75.3%. An apparent internal side reaction occurred. For GPE-80, the coulombic efficiency was very stable, staying at approximately 99%. This stability indicates that the side reaction is minimal, and less energy is lost during charging and discharging, which is consistent with the electrochemical window test results. The electrochemical window of GPE-80 is greater than the operating voltage of NCM811 of 4.3 V. Therefore, GPE-80 has few side reactions and a high coulombic efficiency. In contrast, the oxidation potential of the liquid electrolyte was lower than 4.3 V for NCM811 as shown in Figure 5a. Therefore, during the charging and discharging process, it will continue to decompose. Therefore, its coulombic efficiency is low and changes greatly.

Figure 6c shows the rate performance of GPE-80 at different discharge current densities. The results show that the discharge capacities at 0.1 C, 0.2 C, 0.5 C, and 1.0 C are 191.9, 173.4, 149.3, 125.2, and 139.7 mAh/g, respectively. Obviously, it has good charge and discharge rate performance. Figure 6d shows the charge and discharge profiles of GPE-80 at different cycles. The results show that it is a typical charge and discharge curve of NCM cathode material, and has an inclined discharge platform. The specific charge capacity and the specific discharge capacity decreased gradually with increasing number of cycles, which is consistent with the cycle test results.

**Figure 7:**
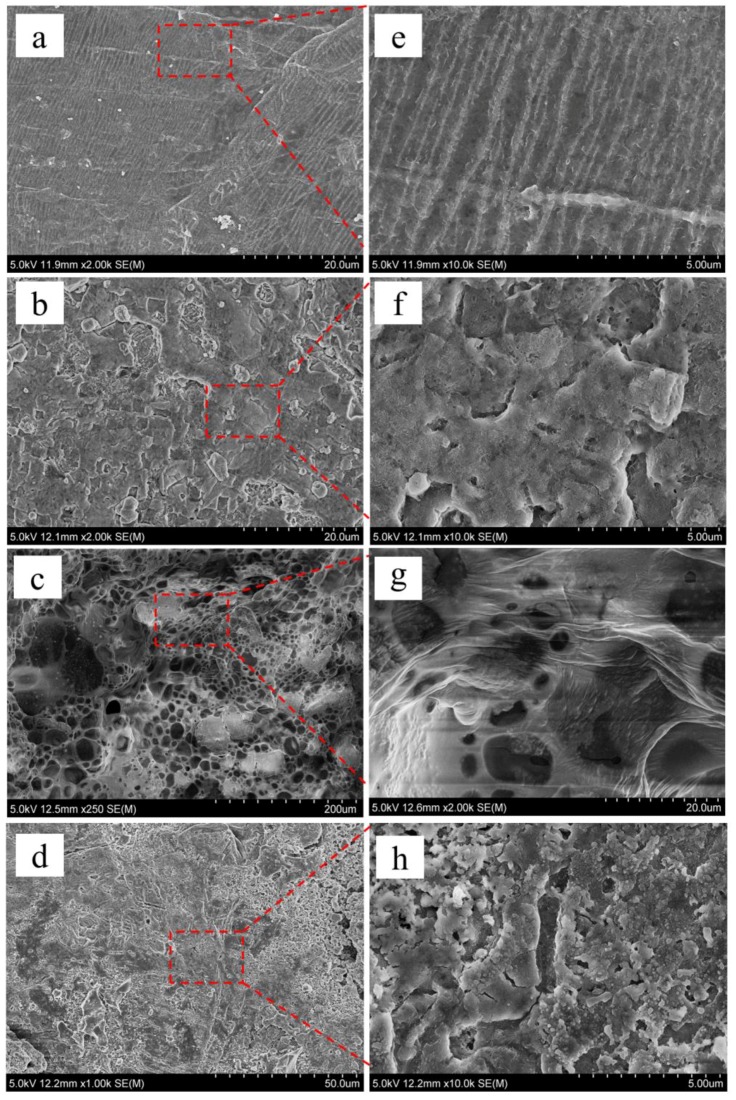
SEM images of lithium negative electrode: (**a**) Fresh lithium tablets, (**b**) in Li|GPE-80|Li cell after 1000 cycles, (**c**) in liquid electrolyte after 80 cycles, (**d**) and in GPE-80 full cell after 80 cycles. (**e**–**h**) are magnified images of selected areas in (**a**–**d**), respectively.

To further study the effect of an electrolyte on the suppression of dendrites, we performed SEM tests on the lithium anodes, and results are shown in Figure 7. As shown in Figure 7a,e, the microstructure of the surface of fresh lithium metal is rough and uneven, and oriented in a gully-like structure. Figure 7b,f are SEM images of lithium negative electrodes in Li|GPE-80|Li cells after 1000 cycles. Obviously, the gully-like structure disappears, indicating that during the cycle of the battery, the lithium stripping/plating fills the original gullies. Spherical lithium can be observed on the surface. According to statistics, the minimum, maximum, and average diameters are 0.65, 3.76, and 1.84 µm, respectively.

Figure 7c,g are SEM images of lithium electrodes in liquid electrolytes after 80 cycles. The surface is very uneven and has large particles. The minimum, maximum, and average diameters are 10.94, 43.17, and 28.12 µm, respectively. Figure 7d,h shows SEM images of lithium electrodes in GPE-80 full cells after 80 cycles. The surface is relatively uniform and flat, and the diameter of the particles on the surface is small. Through statistics, the minimum, maximum, and average diameters are 0.13, 0.45, and 0.28 µm, respectively.

Obviously, the spherical lithium particles in the GPE80 electrolyte are much smaller than those in the liquid electrolyte. GPE80 can suppress the growth of lithium dendrites.

**Figure 8:**
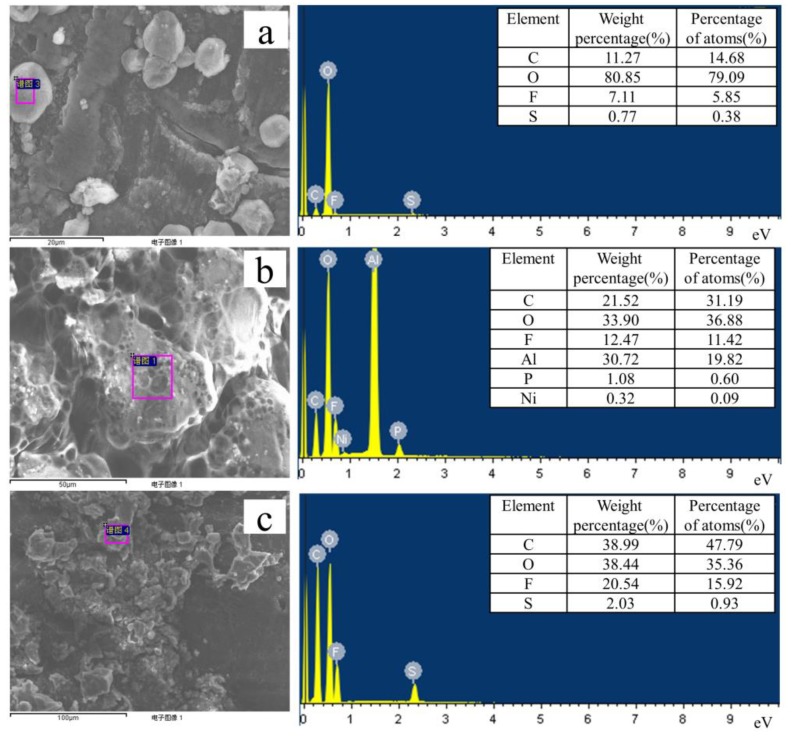
EDS tests results of lithium electrodes: (**a**) In Li|GPE-80|Li cell after 1000 cycles, (**b**) in liquid electrolyte after 80 cycles, and (**c**) in a GPE-80 full cell after 80 cycles.

To study the SEI (solid electrolyte interphases) on the lithium electrode and the dissolution of ions on a positive NCM, we performed semi quantitative EDS elemental analysis, and the results are shown in Figure 8. The surface elemental analysis of the lithium-negative electrode in Li|GPE-80|Li cells after 1000 cycles is shown in Figure 8a. The results show that the contents of C, O, F, and S are 11.27, 80.85, 7.11, and 0.77 wt. %, respectively. Figure 8c shows that for the lithium electrode in the GPE-80 full cell after 80 cycles, the C, O, F, and S contents are 38.99, 38.44, 20.54, and 2.03 wt. %, respectively. Obviously, symmetrical cells and full cells have the same element type, but differ in composition.

Element analysis of the lithium anode in liquid electrolyte after 80 cycles is shown in Figure 8b. The results show that the contents of C, O, F, Al, P, and K are 21.52, 33.90, 12.47, 1.08, and 0.32 wt. %, respectively. Comparing the elemental analysis results in Figure 8b,c reveals that the liquid electrolyte is seriously decomposing during cycling. The presence of P on the negative electrode demonstrates the lithium salt LiPF_6_ decomposes and is deposited as a precipitate on the surface of the lithium electrode. The presence of Al demonstrates the occurrence of corrosion of the Al foil. The presence of Ni indicates the dissolution of Ni from the positive electrode NCM811 material.

For GPE80, no elements such as Al, P, and Ni are detected on the lithium negative electrode. This further proves its high electrochemical stability, and ability to be used with high-voltage cathode materials.

## 4. Conclusions

A sulfolane plasticized gel electrolyte was prepared by in situ photocuring. The effect of the sulfolane content on the ionic conductivity of the polymer gel electrolytes was researched. Through curve fitting, we found that the ionic conductivity variation trend was in good agreement with the exponential function model. And the ionic conductivity of GPE increases faster when the sulfolane content is higher than 50%. This provides inspiration for the preparation of high ionic conductivity GPE. Through curve fitting, the activation energy was inversely proportional to the sulfolane content. That indicates that the lower the activation energy is, the easier Li+ migrates. For the sulfolane content of 80% in GPE-80 (19.5 kJ/mol), the activation energy is closer to conventional liquid electrolyte (9.5 kJ/mol). Simultaneously, its ionic conductivity and electrochemical window reached 0.64 mS/cm and 5.86 V, respectively. The battery performance test showed that GPE-80 has a better specific discharge capacity and cycling performance than a liquid electrolyte. After 140 cycles, the specific discharge capacity of GPE-80 was maintained at 96.1 mAh/g, and the capacity retention rate reached 54.3%. SEM results indicated that GPE-80 can suppress the growth of lithium dendrites. The EDS test shows that GPE-80 can inhibit the dissolution of metal ions in the cathode material, and is better than conventional liquid electrolyte. In summary, such a high oxidation potential allows it to match most existing high voltage cathode materials, and far exceed conventional liquid electrolytes (4.1 V), indicating that sulfolane-plasticized PEGDA polymer is a high-voltage gel electrolyte with excellent electrochemical stability.

## Supplementary Materials

The following are available online at https://www.mdpi.com/2073-4360/11/8/1306/s1, Figure S1: (a) Nyquist plots of GPEs with different content of sulfolane at 30 °C; Partial amplification of (a) with the different contents of sulfolane: (b) 20 wt. % and 30 wt. %, (c) 40 wt. % and 50 wt. %. (d) from 60 wt. to 90 wt. %, Figure S2: Figure S2. Nyquist plots of different GPEs under variable temperature conditions: (a) GPE-0, (b) GPE-20, (c) GPE-40, (d) GPE-60, (e) GPE-80.
