# Supplementary material for: High-Voltage Sulfolane Plasticized UV-Curable Gel Polymer Electrolyte
Source: Polymers (Basel). 2019 Aug 4;11(8):1306. doi: 10.3390/polym11081306 (PMC6722775; doi:10.3390/polym11081306)
Supplement: Supplementary file 1 [file polymers-11-01306-s001.pdf]

# Supporting Information

## High-voltage sulfolane plasticized UV-curable gel polymer electrolyte

***ShiQi Wang, Chun Wei*, WenWen Ding, LinMin Zou, YongYang Gong*, YuanLi Liu, LiMin Zang and Xu Xu***

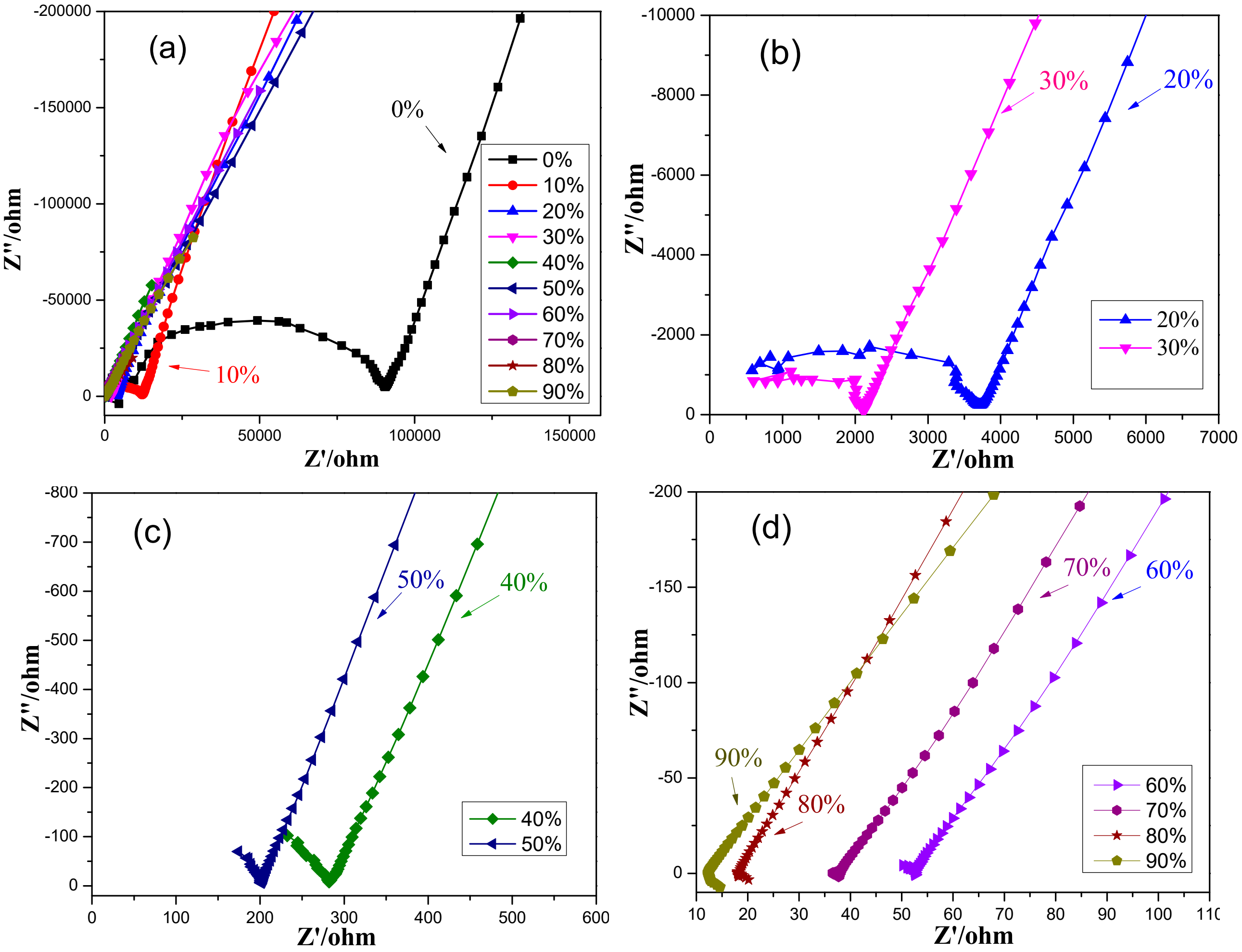


Figure S1. (a) Nyquist plots of GPEs with different content of sulfolane at 30 °C; Partial amplification of (a) with the different contents of sulfolane: (b) 20 wt. % and 30 wt. %, (c) 40 wt. % and 50 wt. %. (d) from 60 wt. to 90 wt. %.

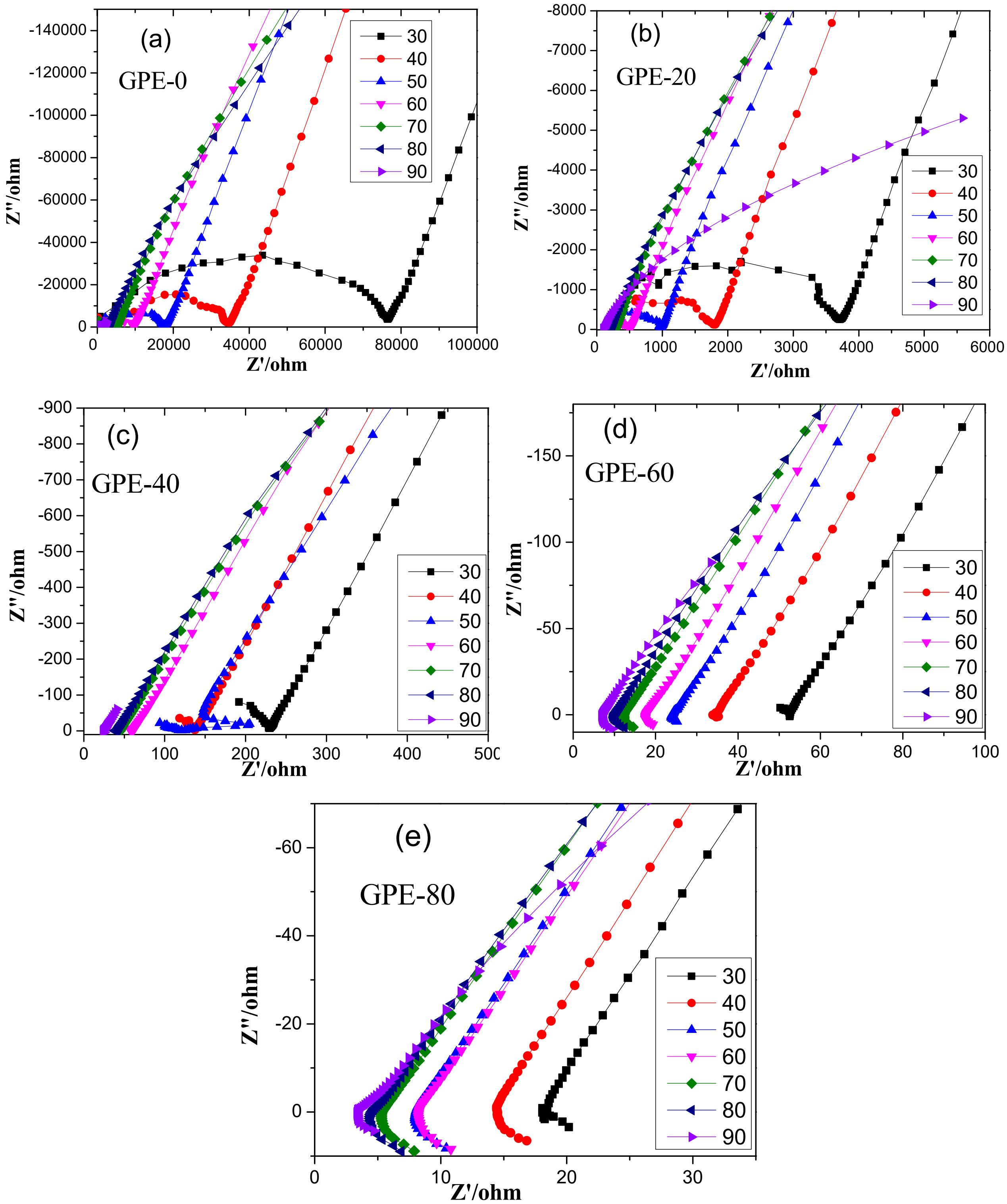


Figure S2. Nyquist plots of different GPEs under variable temperature conditions：(a) GPE-0, (b) GPE-20, (c) GPE-40, (d) GPE-60, (e) GPE-80.
